# Photoluminescence Excitation Engineering of Gold Nanoparticle-Decorated Zinc Oxide Nanoflowers for Efficient Visible Photocatalytic Water Treatment

**DOI:** 10.1021/acsomega.6c03907

**Published:** 2026-06-16

**Authors:** Siwaporn Khemphet, Sayan Pudwat, Nattasamon Petchsang, Yong-Hoon Kim, Tanakorn Osotchan, Pairote Jaideaw, Nopporn Poolyarat, Rawat Jaisutti

**Affiliations:** † Department of Physics, Faculty of Science and Technology, 65100 Thammasat University, Pathum Thani 12120, Thailand; ‡ Research Unit in Innovative Sensors and Nanoelectronic Devices, Thammasat University, Pathum Thani 12120, Thailand; § Department of Materials Science, Faculty of Science, 54775 Kasetsart University, Bangkok 10900, Thailand; ∥ School of Advanced Materials Science and Engineering, 35017 Sungkyunkwan University, Suwon 16419, Korea; ⊥ Department of Physics, Faculty of Science, 98842 Mahidol University, Bangkok 10400, Thailand; # Physics and General Science Program, Faculty of Science and Technology, 364517 Nakhon Ratchasima Rajabhat University, Nakhon Ratchasima 30000, Thailand; ∇ 93823 Thailand Institute of Nuclear Technology, Nakhon Nayok 26120, Thailand

## Abstract

Plasmonic metal–semiconductor heterostructures represent an effective strategy for enhancing visible-light-driven photocatalysis and suppressing charge recombination in wide-bandgap oxides. Here, we report the synthesis of hierarchical Au nanoparticle-decorated ZnO nanoflowers (Au/ZnO NFs) and systematically investigate their plasmon-assisted photocatalytic mechanisms. Structural and compositional analyses confirm that Au nanoparticles are uniformly distributed on the ZnO NF surface without disrupting the wurtzite crystal structure. UV–Vis diffuse reflectance spectroscopy demonstrates enhanced visible-light absorption induced by the localized surface plasmon resonance of Au nanoparticles, while the intrinsic band gap of ZnO remains unchanged. Photophysical investigations using steady-state photoluminescence (PL), PL excitation, and time-resolved PL demonstrate that Au decoration significantly suppresses both near-band-edge and defect-related recombination. This suppression arises from plasmon-induced hot-electron generation, efficient interfacial electron transfer, and electron trapping at the Au–ZnO interface, resulting in prolonged carrier lifetimes and enhanced charge separation. These effects promote the generation of reactive oxygen species under visible-light irradiation. Consequently, the Au/ZnO NFs exhibit enhanced visible-light-driven photocatalytic degradation of methylene blue, achieving nearly complete dye removal within a short irradiation time of 30 min. This study proposes a mechanistic understanding of plasmon-enhanced charge-transfer processes in Au/ZnO heterostructures and establishes a promising strategy for designing efficient visible-light-driven photocatalysts for catalytic wastewater treatment.

## Graphical abstract

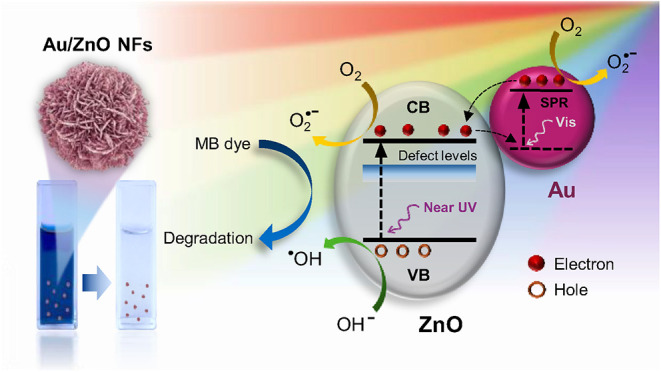

## 1 Introduction

Rapid industrialization and urbanization are major contributors to the increasing release of organic pollutants into aquatic environments.^1^ Among these pollutants, synthetic dyes such as methylene blue (MB) are frequently detected in industrial wastewater because of their widespread use in textile, paper, leather, and pharmaceutical industries.^2,3^ MB is a cationic dye that is highly water-soluble and resistant to biodegradation. It has been reported to exhibit mutagenic and potentially carcinogenic effects, posing serious risks to aquatic ecosystems and human health.^4,5^ Thus, the development of efficient and sustainable technologies for dye removal and wastewater treatment is of great significance.

Semiconductor-based photocatalysis has emerged as a promising strategy for water treatment owing to its ability to utilize light energy to induce oxidative degradation of organic contaminants.^6–9^ Zinc oxide (ZnO), a wide-bandgap semiconductor with high chemical stability, environmental friendliness, and low cost, has been extensively studied as a photocatalyst.^10,11^ In particular, hierarchical ZnO nanostructures, such as nanoparticles,^12,13^ nanowires,^14^ and nanotubes,^15^ offer large surface areas and abundant active sites, which are beneficial for photocatalytic reactions. In addition, the photocatalytic degradation efficiency of ZnO can also be further enhanced through the formation of composites with carbon-based materials,^16,17^ and other metal oxide/sulfide semiconductors.^18,19^ However, despite these advantages, the practical application of ZnO remains limited by its absorption primarily in the ultraviolet (UV) region, and by the rapid recombination of photogenerated electron–hole pairs. To overcome these limitations, metal–semiconductor heterostructures have attracted considerable attention as an effective approach for enhancing light-driven photocatalysis and suppressing charge recombination in wide-bandgap oxides.^20^ Noble metal nanoparticles exhibit localized surface plasmon resonance (LSPR) upon light illumination, which enables the generation of energetic (hot) electrons under resonant excitation.^21,22^ When decorated on ZnO nanostructures, plasmonic metals can facilitate hot-electron injection, enhance interfacial charge transfer, and serve as electron sinks.^23–25^ Therefore, the decoration of ZnO nanostructures with noble metal nanoparticles can significantly improve charge separation and photocatalytic efficiency under UV and visible-light irradiation.

For effective light harvesting, understanding photoluminescence (PL) processes and defect states plays a crucial role in regulating charge-carrier dynamics in semiconductor photocatalysts.^26,27^ Defect levels within ZnO, such as zinc interstitials and oxygen vacancies, strongly influence charge-carrier recombination pathways and visible emission behavior.^28^ Engineering these excitation–emission pathways through plasmonic modification provides an effective route to regulate carrier relaxation, suppress charge-carrier recombination, and enhance reactive oxygen species generation. However, despite growing interest in plasmon-enhanced ZnO photocatalysts, a comprehensive understanding of how metal nanoparticles modulate photoluminescence excitation (PLE) behavior, defect-mediated recombination, and charge-transfer kinetics in hierarchical ZnO nanostructures remains limited. PLE engineering provides a powerful approach to reveal how metal nanoparticles modify defect-mediated excitation pathways in metal oxide semiconductors, offering mechanistic insight beyond conventional structural, optical absorption, and photocatalytic performance analyses.^29^

In this work, we report the synthesis of hierarchical Au nanoparticle-decorated ZnO nanoflowers (Au/ZnO NFs) via a low-temperature solution process and systematically investigate their plasmon-assisted photocatalytic mechanisms. By integrating structural characterization with PL, PLE, and time-resolved photoluminescence (TRPL) analyses, the excitation pathway and interfacial charge-transfer mechanisms of ZnO and Au/ZnO are investigated. The photocatalytic performance of the Au/ZnO NFs is evaluated through the degradation of MB under visible-light irradiation, demonstrating significantly enhanced activity with nearly complete dye degradation within 30 min. This work provides mechanistic insight into plasmon-enhanced charge-transfer processes and PLE engineering in Au/ZnO heterostructures and establishes a promising strategy for designing efficient visible-light-driven photocatalysts for catalytic wastewater treatment.

## 2 Experimental Section

### 2.1 Synthesis of ZnO NFs

Zinc acetate dihydrate (Zn­(CH_3_COO)_2_·2H_2_O, ≥98%), sodium citrate dihydrate (Na_3_C_6_H_5_O_7_·2H_2_O, ≥99%), sodium hydroxide (NaOH, ≥98%), gold­(III) chloride hydrate (HAuCl_4_, 99.99%), and ascorbic acid (C_6_H_8_O_6_) were purchased from Sigma-Aldrich. All chemical reagents were used as received without further purification. ZnO NFs were synthesized by a facile chemical process. Initially, 0.1 M of zinc acetate dihydrate and 0.24 M of sodium citrate dihydrate were dissolved in 30 mL of deionized (DI) water under vigorous stirring at 40 °C for 20 min to form a transparent solution. Then, 0.5 M of sodium hydroxide was added to the above solution and stirred continuously at 40 °C for 30 min to form a white suspension and naturally cooled down to room temperature. The resulting precipitation was then washed by centrifugation with DI water and ethanol three times to remove impurities. Finally, the white powder of ZnO NFs was obtained after being fully dried in an oven at 60 °C for 12 h.

### 2.2 Synthesis of Au-Decorated ZnO NFs

Au nanoparticles were decorated on the surface of ZnO NFs through a chemical reduction method. In a typical synthesis, 100 mg of as-prepared ZnO NFs was dispersed in 20 mL of DI water and magnetically stirred at 80 °C for 20 min to obtain a homogeneous suspension. Separately, an aqueous solution containing gold­(III) chloride hydrate (5 wt % relative to ZnO NFs) and 0.1 M sodium citrate dihydrate was prepared by stirring at room temperature for 10 min. This gold precursor solution was then added dropwise into the ZnO suspension and continuously stirred at 80 °C for 10 min, during which the mixture gradually turned from milky white to light gray. Subsequently, 0.01 M ascorbic acid was introduced as a reducing agent to convert Au^3+^ to Au^0^, followed by stirring for an additional 30 min. The resulting suspension was washed alternately with DI water and ethanol three times via centrifugation, and the precipitate was dried in an oven at 60 °C for 12 h. A pink powder of Au/ZnO NFs was finally obtained. To investigate the effect of Au content on photocatalytic performance, the wt % of HAuCl_4_ relative to ZnO NFs was systematically varied at 3, 5, 10, and 15 wt %, denoted as Au3/ZnO, Au5/ZnO, Au10/ZnO, and Au15/ZnO, respectively.

### 2.3 Characterization of ZnO and Au/ZnO NFs

The morphology and surface features of ZnO and Au/ZnO nanostructures were examined using scanning electron microscopy (SEM, FEI Quanta 450). Energy-dispersive X-ray spectroscopy (EDS) was employed to analyze the elemental composition and confirm the uniform distribution of Au nanoparticles on the ZnO surface. The crystalline phase and structural properties were determined by X-ray diffraction (XRD, Bruker D8 Advance) using Cu Kα_1_ radiation (λ = 1.54060 Å) in the 2θ range of 20°–80°. The nanostructural characteristics were further investigated by field-emission transmission electron microscopy (FETEM, JEOL JEM-3100F). The molecular structure of pristine and Au/ZnO NFs was analyzed using Raman spectroscopy (HORIBA, XploRA PLUS). X-ray photoelectron spectroscopy (XPS, Kratos AXIS Ultra DLD) was used to identify the chemical states and bonding configurations of the elements present on the surface. Optical absorption spectra were obtained using UV–Vis spectroscopy (Shimadzu, UV-2600) to study the light absorption behavior and estimate the optical band gap energy. In addition, PL, PLE, and TRPL measurements (HORIBA, FluoroMax Plus) were performed to investigate the charge recombination dynamics and defect-related emissions of the ZnO and Au/ZnO nanostructures.

### 2.4 Photocatalytic Activities of ZnO and Au/ZnO NFs

The photocatalytic activities of ZnO and Au/ZnO NFs were evaluated through the degradation of MB under visible-light irradiation. A stock MB solution with a concentration of 15 μM was prepared by dissolving the dye in 1 L of DI water. Then, 0.01 g of Au/ZnO photocatalyst was added to 40 mL of the stock solution and magnetically stirred in the dark for 30 min to establish adsorption–desorption equilibrium between the dye molecules and the catalyst surface. Subsequently, the reaction suspension was irradiated under visible light (50 W low voltage halogen lamp, Philips) for 1 h to initiate the photocatalytic degradation. At specific time intervals, 2 mL of the reaction solution was extracted and centrifuged to remove the catalyst particles. The absorbance of the clear supernatant was measured using a UV–Vis spectrophotometer (Thermo Scientific, Genesys 10 Series) to monitor the change in the residual MB concentration over time. The normalized concentration (*C_t_*/*C*_0_) during MB photodegradation was calculated from the ratio of the absorbance at time *t* (*A_t_*) to the initial absorbance (*A*_0_) measured at 664 nm, assuming the Beer–Lambert law.^30,31^

## 3 Results and Discussion

### 3.1 Surface Morphology and Crystal Structure of ZnO and Au/ZnO NFs

As described, ZnO and Au/ZnO NFs were synthesized using a low-temperature solution process. SEM analysis shows that the pristine ZnO NFs exhibit a distinct NF morphology, consisting of radially assembled nanosheets that form three-dimensional hierarchical structures, as shown in Figure 1a. The size of NFs typically ranged from 2–4 μm in diameter, while each nanosheet displays a smooth surface and well-defined edges, reflecting uniform crystal growth during the synthesis process. This hierarchical assembly provides a large surface area and abundant active sites, which are advantageous for photocatalytic reactions. Upon decoration with Au nanoparticles, SEM images reveal that the overall structure remains identical to that of pristine ZnO NFs. The preservation of the ZnO framework suggests that the Au nanoparticle decoration process does not disrupt hierarchical morphology.

**Figure 1:**
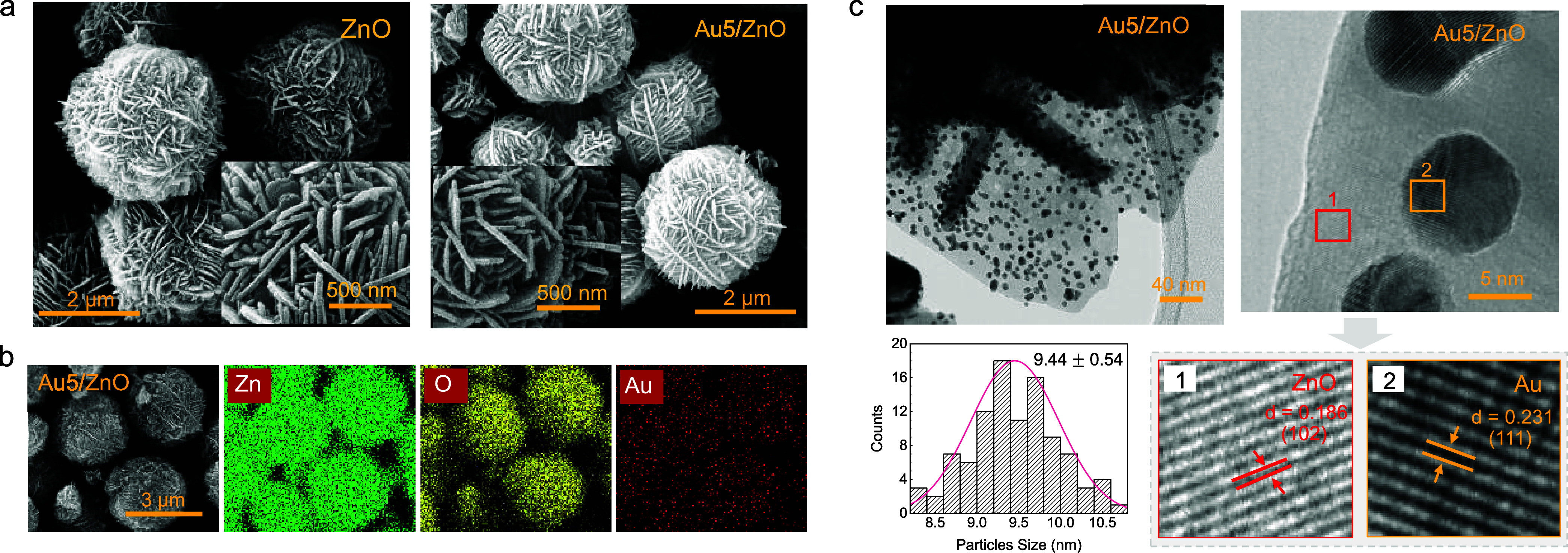
Surface morphology and elemental characterization of ZnO and Au/ZnO NFs. (a) SEM images of pristine ZnO and Au5/ZnO NFs. (b) Elemental mapping of the Au5/ZnO NFs. (c) TEM images of Au5/ZnO NFs. High-resolution TEM images reveal lattice fringes corresponding to ZnO and Au. The inset in (c) shows the particle size distribution of Au nanoparticles decorated on the ZnO surface.

To confirm the successful incorporation of Au nanoparticles on the ZnO NFs, EDS was performed, and the corresponding results are shown in Figure S1. The EDS spectra of pristine ZnO NFs show characteristic signals for Zn and O with no detectable impurities. After Au nanoparticle decoration, additional elemental peaks corresponding to Au atoms appear in the EDS spectrum. The presence of these peaks confirms the successful nucleation and attachment of Au nanoparticles to the ZnO surface. Elemental mapping, shown in Figure 1b, further supports the uniform distribution of the Au nanoparticles. The Zn and O signals are consistently mapped across the NF structure, while the Au mapping reveals well-dispersed nanoparticles localized on the NFs.

TEM analysis was conducted to clarify the structural characteristics of the Au/ZnO NFs. Unlike the SEM observations, where Au nanoparticles could not be distinguished on the ZnO NFs, the TEM images provide clear evidence of Au nanoparticles anchored on the ZnO, as shown in Figure 1c. The ZnO structures appear as layered or sheet-like domains with defined structural ordering, while the Au nanoparticles are visible as dark, spherical dots distributed across the ZnO surface. The particle size is predominantly in the range of 9–10 nm. High-resolution TEM images reveal the crystalline nature of both components. The lattice fringe spacing of 0.186 nm in the ZnO region corresponds to the (102) planes of hexagonal wurtzite ZnO, while the lattice spacing of 0.231 nm observed in the Au nanoparticles matches well with the (111) planes of face-centered cubic (FCC) Au. Additionally, Au nanoparticles are uniformly dispersed and form a distinct, intact interface with the ZnO matrix at all loading levels, consistent with the properties observed across all Au/ZnO samples (Figure S2). Higher Au-loading samples exhibit increased Au content, as confirmed by the EDS results shown in Figure S1. These observations confirm the successful formation of well-integrated Au nanoparticles decorating the surfaces of ZnO NFs.

The crystalline structures of pristine ZnO and Au/ZnO NFs were examined using XRD, and the results are shown in Figure 2a. The XRD pattern of the pristine ZnO sample corresponds to the crystallographic planes of hexagonal wurtzite ZnO (JCPDS No. 36–1451). For the Au/ZnO samples, the XRD patterns retain all characteristic ZnO peaks without observable peak shifts, confirming that Au decoration does not alter the intrinsic crystal structure of ZnO. In addition to the ZnO peaks, weak diffraction signals attributable to metallic Au are also detected at around 38.3° and 44.5°, corresponding to the (111) and (200) planes of FCC Au nanoparticles (JCPDS No. 04–0784).^32^ Additional diffraction peaks are also observed at a 2θ value of 64.7°, which are associated with the (220) planes of Au, particularly for the higher Au loadings (10% and 15% AuHCl_4_). Although these peaks are relatively low in intensity due to the small size and low loading amount of Au, their presence clearly verifies the successful incorporation of Au crystallites onto the ZnO surface.

**Figure 2:**
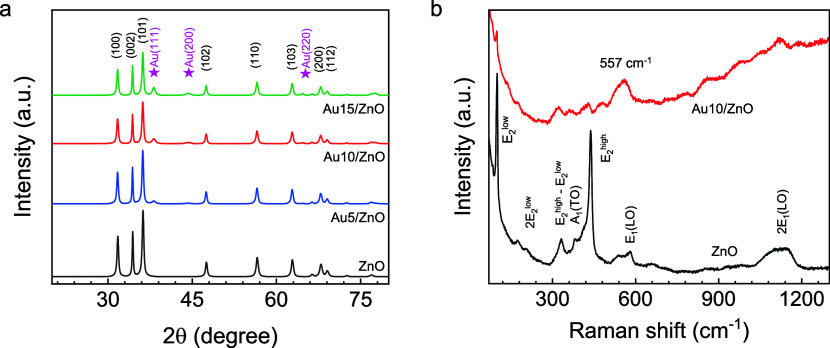
Structural characterization of pristine ZnO and Au/ZnO NFs: (a) XRD patterns and (b) Raman spectra.

Raman spectroscopy for the pristine ZnO and Au/ZnO NFs measured at room temperature is shown in Figure 2b. The pristine ZnO NFs exhibit the characteristic vibrational modes of hexagonal wurtzite ZnO, with Raman peaks observed at approximately 99, 205, 330, 382, 438, 580, and 1117 cm^–1^ under 532 nm excitation.^33,34^ The intense peaks at 99 and 438 cm^–1^ correspond to the E_2_^low^ and E_2_^high^ modes, which arise from the zinc and oxygen sublattice vibrations, respectively.^35,36^ The peaks at 205 and 330 cm^–1^ are associated with the second-order nonpolar E_2_ mode (2E_2_^low^), and the E_2_^high^–E_2_^low^ multiphonon process, respectively. The peak at 382 cm^–1^ is attributed to the transverse optical (TO) component for the A_1_ mode (A_1_(TO)). The peak at 580 cm^–1^ corresponds to the longitudinal optical (LO) component of the E_1_ mode (E_1_(LO)), while the band at 1117 cm^–1^ is attributed to the second-order Raman scattering. These well-defined Raman modes confirm the preservation of the wurtzite ZnO NF structure. For the Au/ZnO samples, the overall Raman features remain consistent with the wurtzite ZnO phase, although a broader background is observed after Au decoration. A slight red shift (from ∼438 to ∼436 cm^–1^), accompanied by reduced intensity and peak broadening of the E_2_^high^ mode, suggests subtle modifications of Zn–O lattice vibrations, and interfacial strain effect associated with Au–ZnO interactions.^35,37^ These structural modifications may facilitate charge separation and suppress electron–hole recombination, thereby contributing to enhanced photocatalytic activity.

XPS analysis was carried out to investigate the chemical states and surface composition of the Au/ZnO nanocomposite. The XPS spectra of pristine ZnO and Au/ZnO NFs are represented in Figure 3. The survey spectrum shown in Figure 3a confirms the presence of Zn, O, and Au elements, consistent with the EDS and XRD findings. Figure 3b illustrates the high-resolution of Zn 2p spectra. Two distinct peaks are observed at ∼ 1020.7 eV and ∼1043.8 eV, corresponding to the Zn 2p_3/2_ and Zn 2p_1/2_ states, respectively. The energy separation of 23.1 eV between these peaks is characteristic of the Zn^2+^ oxidation state in the wurtzite ZnO structure.^38^ Figure 3c shows the fine-scan Au 4f and Zn 3p for the Au/ZnO NFs. The Au 4f_7/2_ and Au f_5/2_ peaks appear at ∼83.3 eV and ∼87.0 eV, respectively, matching well with the binding energies of metallic Au^0^.^39^ Moreover, the Zn 3p signal is observed to overlap with the Au 4f region, appearing at ∼88.5 eV (Zn 3p_3/2_) and ∼91.3 eV (Zn 3p_1/2_). The concurrent detection of Zn 3p and Au 4f signals demonstrates that the Au nanoparticles are highly dispersed and of sufficiently small size to prevent full attenuation of the underlying Zn signal. For O 1s analysis, the high-resolution O 1s spectra of pristine ZnO and Au/ZnO NFs are shown in Figure 3d,e, respectively. The O 1s peak can be deconvoluted into three primary components. The main peak located at ∼529.9 eV is attributed to lattice oxygen (O_L_) bound to Zn^2+^ within the wurtzite ZnO structure. A secondary component appearing at ∼531.3 eV corresponds to the vicinity of oxygen vacancies (V_o_) in the ZnO lattice (O_V_). The third peak, observed at ∼532.9 eV, is associated with chemisorbed oxygen species (O_C_).^40,41^ The relative intensity of the O_V_/O_L_ component decreases slightly in the Au/ZnO sample, indicating that Au nanoparticle decoration modulates the concentration of oxygen vacancies in ZnO. This reduction may arise from the passivation of defect sites by the Au nanoparticles, electron withdrawal from vacancy states, or stabilization of the ZnO surface during the Au decoration process.

**Figure 3:**
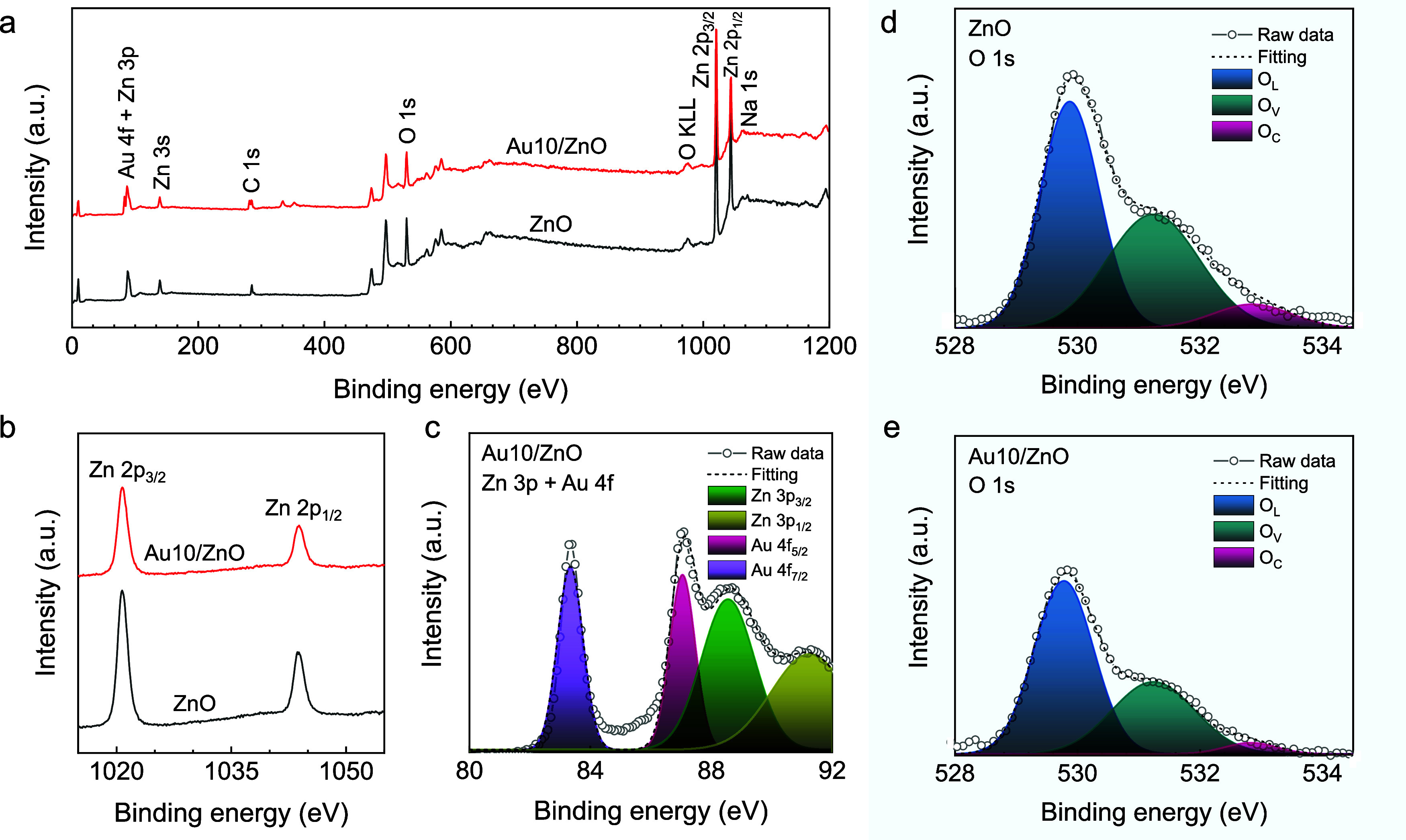
XPS spectra of pristine ZnO and Au/ZnO NFs. (a) Survey XPS spectra. High-resolution core-level spectra of (b) Zn 2p, (c) Zn 3p/Au 4f, and O 1s for (d) pristine ZnO and (e) Au/ZnO NFs.

### 3.2 Optical Properties of Au/ZnO NFs

The light absorption characteristics of pristine ZnO and Au/ZnO NFs were investigated using UV–Vis diffuse reflectance spectra, as shown in Figure 4a. The pristine ZnO NFs exhibit a sharp absorption edge in the near-UV region at approximately 380 nm, which is consistent with the intrinsic band-to-band absorption of ZnO. In addition, the ZnO sample shows minimal absorption in the visible range, caused by a low concentration of defect states, as observed by the O 1s XPS analysis.^42^ Upon decoration with Au nanoparticles, the Au/ZnO NFs display an additional broad reflected band in the visible region, centered around ∼520 nm, which is attributed to the surface plasmon resonance (SPR) of metallic Au nanoparticles.^34,42^ Notably, the SPR peak position of the Au/ZnO NFs with different Au loading (5–15 wt %) remains nearly unchanged. The optical band gaps of the samples were estimated using Tauc plots derived from the Kubelka–Munk transformed reflectance data.^43^ Assuming a direct allowed transition for ZnO, the *(F­(R)­hv)* ^2^ vs *hv* plots (Figure 4b) show that pristine ZnO possesses an optical band gap of approximately 3.25 eV. A slight reduction in the optical band gap is observed for the Au/ZnO NFs (∼3.23 eV), which may be attributed to Au-induced defect states and interfacial electronic interactions rather than intrinsic modification of the ZnO crystal structure. In addition, the LSPR of Au nanoparticles enhances visible-light absorption, leading to increased sub-bandgap optical transitions.

**Figure 4:**
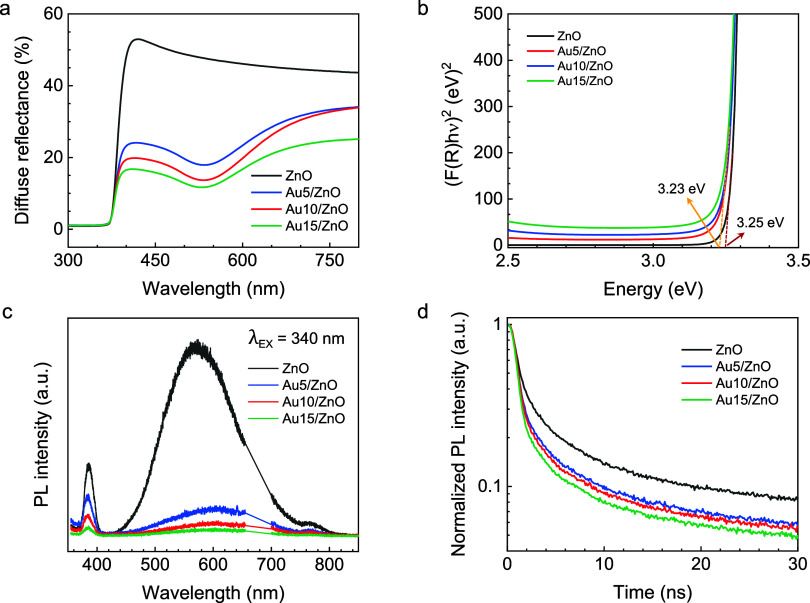
(a) UV–vis diffuse reflectance spectra of pristine ZnO and Au/ZnO NFs, and (b) corresponding optical band gap determination using the Tauc plot. (c) PL spectra and (d) TRPL decay profiles of pristine ZnO and Au/ZnO NFs.

To understand insight into the recombination behavior of charge carriers in the ZnO and Au/ZnO NFs, room-temperature PL and PLE measurements were carried out. Figure 4c shows the PL spectra of pristine ZnO NFs and Au/ZnO NFs decorated with different amounts of Au nanoparticles. Under 340 nm excitation, the pristine ZnO NFs exhibit a strong near-band-edge (NBE) emission at approximately ∼378–382 nm, along with a broad visible emission band spanning ∼450–600 nm. The narrow UV emission peak is attributed to the radiative recombination of free excitons in ZnO.^34^ In contrast, the broad visible emission band is associated with deep-level defect states in ZnO. The wide visible emission indicates the presence of various defect states, such as zinc interstitials, zinc vacancies, and oxygen vacancies.^44,45^ The strong deep-level emission intensity suggests a relatively high concentration of these defect states in the pristine ZnO NFs. Compared with the pristine ZnO NFs, the Au/ZnO NFs exhibit significant PL quenching in both the NBE and defect-related emission regions, indicating suppressed electron–hole recombination. This quenching effect is attributed to the efficient trapping of photogenerated electrons by the Au nanoparticles, which act as electron sinks at the Au–ZnO interface. A stronger quenching effect is observed with increasing Au nanoparticle loading, suggesting enhanced charge extraction efficiency. In addition, the defect-related PL intensity becomes lower than the NBE emission intensity in the Au/ZnO samples. This behavior is likely due to effective passivation of defect states and rapid charge electron transfer to Au nanoparticles, which suppresses defect-mediated recombination and enhances charge separation.

The TRPL spectra of pristine ZnO and Au/ZnO NFs are shown in Figure 4d. The decay curves were fitted using a biexponential model, yielding two characteristic lifetimes, τ_1_ and τ_2_, which are summarized in Table S1. The fast decay component (τ_1_) is typically associated with the recombination of free or weakly trapped charge carriers through radiative pathways, whereas the slower component (τ_2_) is related to defect- or surface-state-mediated recombination processes, which are predominantly nonradiative in nature.^32,46^ For pristine ZnO, the fast decay component (τ_1_ ≈ 1.12 ns) is associated with rapid recombination of free or weakly trapped charge carriers, while the slower component (τ_2_ ≈ 8.99 ns) corresponds to recombination via defect-related states or surface traps. Upon decoration with Au nanoparticles, τ_1_ shows a slight shorter to ∼1.04 ns, ∼1.03 ns, and ∼0.95 ns for Au/ZnO with 5, 10, and 15 wt % Au loading, respectively. This minor variation suggests that the initial ultrafast relaxation process remains largely unaffected by Au incorporation. In contrast, a pronounced increase in the long lifetime component τ_2_ is observed for the Au/ZnO samples, increasing to ∼10.39 ns for 5 wt % Au and ∼11.07 ns for 10 wt % Au. The prolongation of τ_2_ indicates slow recombination through more defect or surface states and can enhance charge separation efficiency. This behavior can be attributed to electron transfer from ZnO to Au nanoparticles at the Au–ZnO interface, where Au acts as an electron sink, delaying recombination and extending the carrier lifetime. The gradual increase in τ_2_ with higher Au loading further confirms that Au nanoparticles play a crucial role in promoting charge extraction and inhibiting recombination pathways. These TRPL results are fully consistent with the observed PL quenching behavior and support the conclusion that Au decoration effectively improves charge-carrier dynamics, which is beneficial for enhanced photocatalytic performance.

PLE spectroscopy was employed to clarify the excitation pathways responsible for NBE and defect-related emissions in pristine ZnO and Au/ZnO NFs. PLE spectra were recorded by monitoring the emission at 380 nm (NBE) and 575 nm (defect emission), as shown in Figure 5a,b, respectively. When monitoring the 380 nm emission, both pristine ZnO and Au/ZnO samples exhibit a dominant excitation peak at ∼340 nm, which is attributed to intrinsic band-to-band transitions in ZnO, confirming the excitonic origin of the NBE emission. In contrast, when monitoring the 575 nm defect-related emission, pristine ZnO shows a strong excitation peak at ∼380 nm, indicating that visible emission primarily originates from carriers generated by band-edge excitation followed by relaxation into deep-level defect states. No additional high-energy excitation features are observed in ZnO. For the Au/ZnO samples, the PLE spectra monitored at 575 nm retain the excitation peak at ∼380 nm and additionally exhibit a new excitation feature at ∼298 nm, which is absent in pristine ZnO NFs (Figure 5c). The emergence of this additional excitation pathway suggests that Au decoration modifies interfacial electronic states and carrier excitation dynamic through Au-ZnO coupling rather than intrinsic band-edge transitions. Consistently, under fixed excitation at 298 nm, pristine ZnO NFs exhibit a single visible emission band centered at ∼557 nm, whereas Au/ZnO NFs display two emission components at ∼557 and ∼568 nm. The additional ∼568 nm emission may be associated with Au-modified interfacial states or defect-related relaxation pathways induced by strong metal–semiconductor interactions. These Au-induced excitation–emission pathways enhance carrier redistribution and charge separation, consistent with the PL quenching and prolonged TRPL lifetimes, thereby contributing to the enhanced photocatalytic activity of Au/ZnO NFs.

**Figure 5:**
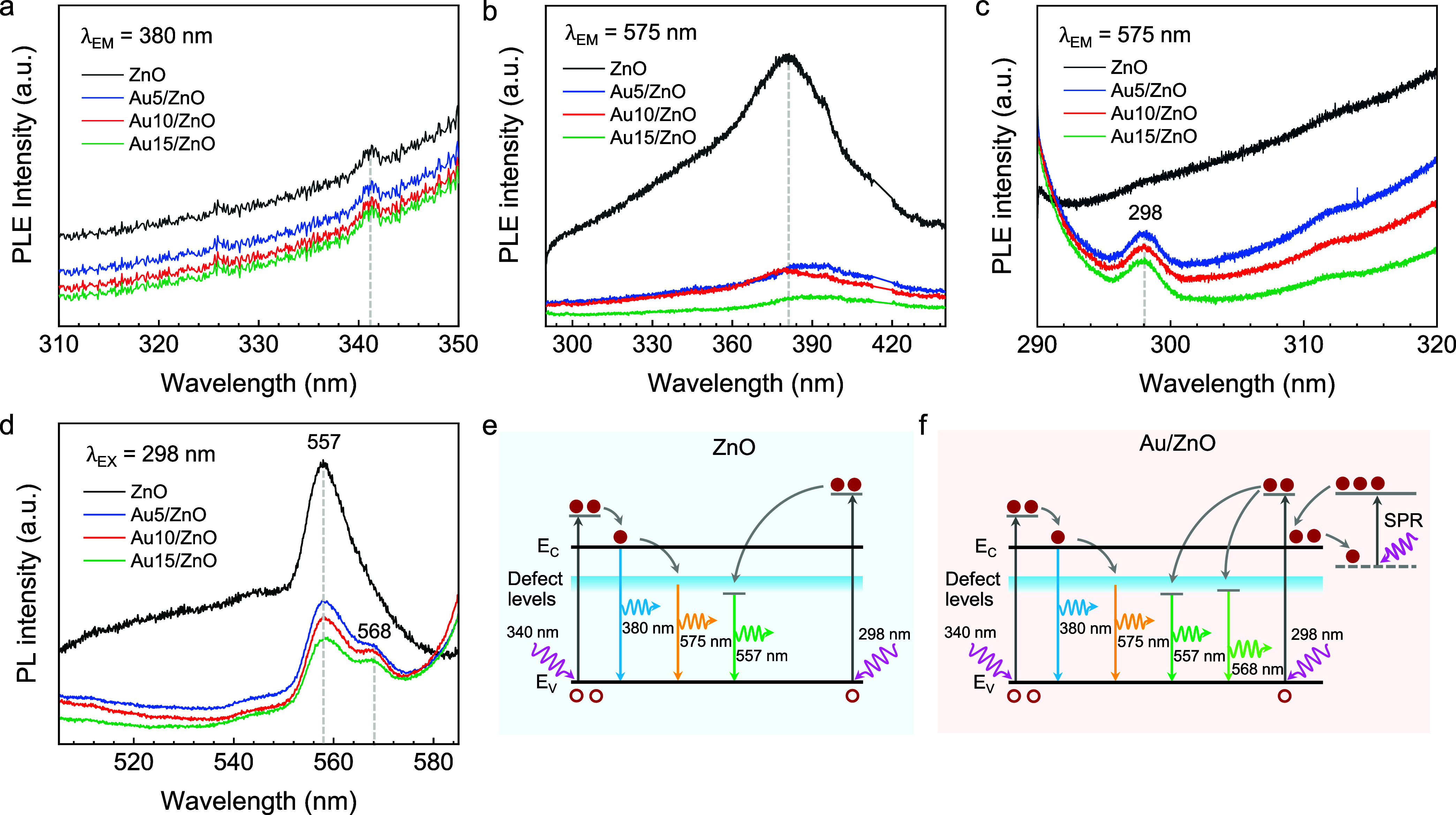
PLE spectra of pristine ZnO and Au/ZnO NFs monitored at emission wavelengths of (a) 380 nm and (b) 575 nm, (c) enlarged view of the shorter-wavelength excitation regions, and (d) PL emission spectra under 298 nm excitation. Schematic illustrations of the charge transfer mechanisms in (e) pristine ZnO, and (f) Au/ZnO heterostructure under photoexcitation.

The underlying electron-transfer mechanism can be modeled based on the energy-band structures of the pristine ZnO and Au/ZnO heterostructure, as illustrated in Figure 5e,f, respectively. For ZnO NFs with a band gap of approximately 3.25 eV, irradiation with 340 nm light promotes electrons from the valence band (VB) to a higher energy state. These photoexcited electrons relax to the conduction band (CB) of ZnO and subsequently recombine with holes at the VB, giving rise to NBE emission. Alternatively, electrons in the CB may be trapped by defect states within the band gap, leading to nonradiative recombination via these defect levels and resulting in a broad visible emission band. For Au/ZnO NFs, photoexcitation occurs not only in ZnO but also through the resonant excitation of localized surface plasmons in the Au nanoparticles, generating high-energy (hot) electrons.^47^ These hot electrons can be injected into the CB of ZnO, resulting in quenching of the NBE emission of Au/ZnO in the PL spectrum. In addition, photogenerated electrons in the CB of ZnO can be transferred to adjacent Au nanoparticles, leading to an increased electron density in the metal. The accumulation of high-density electrons in the Au nanoparticles may populate interfacial defect states, leading to enhanced defect-related emission or additional nonradiative states at approximately 568 nm. Furthermore, the enhanced photocurrent observed in the illuminated I–V characteristics suggests improved interfacial charge transfer and possible plasmon-induced hot-electron contribution in the Au/ZnO heterostructure, as illustrated in Figure S3.

### 3.3 Photocatalytic Activity

Because the decoration of Au nanoparticles on the surface of ZnO NFs enables efficient electron trapping, the resulting composite samples are expected to exhibit enhanced photocatalytic activity. The photocatalytic performance was investigated through the degradation of MB under visible light irradiation. A halogen lamp with a power of 50 W was used as the visible-light source to investigate the photocatalytic performance of the samples, and its emission spectrum is shown in Figure S4. The optical absorption spectra of MB solutions containing pristine ZnO and Au/ZnO NFs under visible-light irradiation are presented in Figure 6a,b, respectively. The photocatalytic reaction was monitored by tracking the decrease in the characteristic absorption peak of MB at a wavelength of 664 nm. No significant MB degradation was observed under visible-light irradiation without a catalyst or in the presence of the catalyst under dark conditions, as shown in Figure S5. In the case of pristine ZnO NFs, a slow reduction in MB absorbance is observed, suggesting relatively poor photocatalytic activity. In contrast, a rapid decrease in absorbance occurs for the Au/ZnO NFs catalyst, demonstrating significantly enhanced visible-light-driven photocatalytic degradation of MB. Figure 6c compares the normalized residual concentration (*C*_t_ */C*_0_) of MB as a function of irradiation time (*t*) for all samples. Here, *C*_0_ and *C*_t_ represent the initial and residual concentration of MB at irradiation time *t*. The photocatalytic activity of pristine ZnO NFs is relatively low, with only approximately 23.8% of the dye degraded after 30 min of visible-light irradiation. However, the MB degradation efficiency increases to ∼77.4% after the introduction of the Au3/ZnO catalyst and proceeds much more rapidly, with nearly complete degradation (∼99.8%) observed for Au-loaded samples with Au contents above 5 wt % (Figure S6). No significant additional enhancement in MB degradation is observed for Au loadings of 10 and 15 wt %. These results indicate that an Au loading of approximately 5 wt % is sufficient to achieve highly efficient visible-light photocatalytic performance under the present experimental conditions.

**Figure 6:**
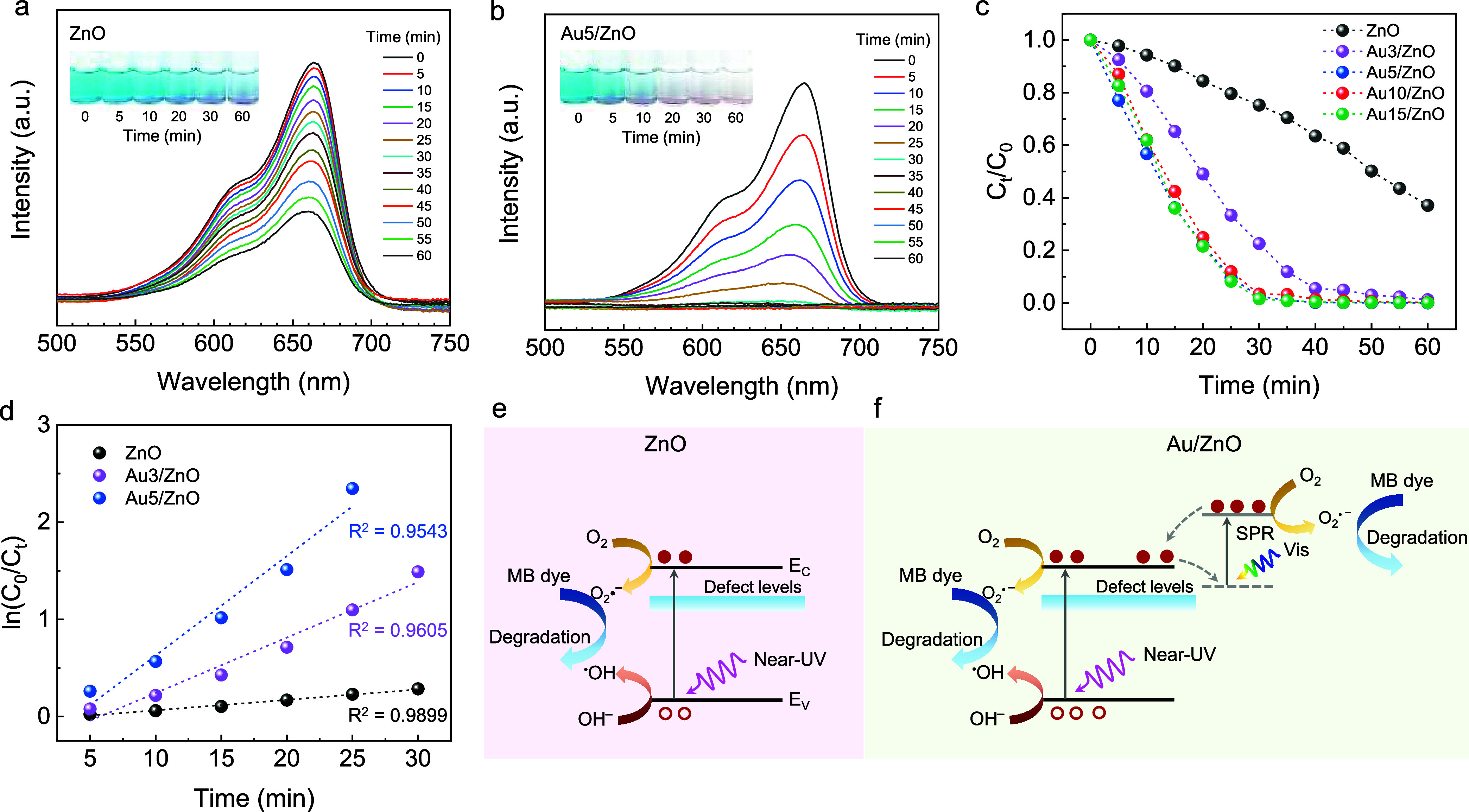
Visible-light-driven photocatalyst performance of pristine ZnO and Au/ZnO NFs for MB degradation. UV–vis absorption spectra of methylene blue solution in the presence of (a) ZnO, and (b) Au5/ZnO photocatalysts. Inset: optical images of MB solutions with catalysts at different irradiation times. (c) Normalized degradation ratio, and (d) corresponding pseudo-first-order kinetic plots as a function of irradiation time. Schematic illustration of the photodegradation mechanisms of (e) pristine ZnO and (f) Au/ZnO photocatalyst under visible-light irradiation.

To further quantify the photocatalytic performance, the degradation kinetics of MB were analyzed by using a pseudo-first-order reaction model.^48^ The kinetic equation can be expressed as ln­(*C*_0_/*C_t_*) *= kt*, where k is the apparent first-order rate constant. The plots of ln­(*C*_0_/*C*_*t*_) vs irradiation time (*t*) for pure MB solutions and in the presence of ZnO and Au/ZnO NFs are shown in Figure 6d. The good linear fitting of these plots confirms that the degradation process follows pseudo-first-order kinetics. The ZnO NFs exhibit a relatively small reaction rate constant (*k* = 0.01 min^–1^), which contributes to their limited photocatalytic activity within the measurement time. In contrast, the Au/ZnO NFs exhibit significantly higher slopes, corresponding to much-enhanced rate constants. Notably, the rate constants of the Au3/ZnO and Au5/ZnO photocatalysts are approximately 5 and 10 times higher than that of pristine ZnO, respectively, demonstrating the strong promotional effect of Au nanoparticles on photocatalytic degradation. The substantial enhancement in the reaction rate is attributed to improved charge separation and accelerated interfacial electron transfer induced by Au nanoparticles, which suppresses charge recombination and promotes the generation of reactive species. These kinetic results are consistent with the PL quenching, prolonged TRPL lifetimes, and modified PLE behavior observed for Au/ZnO NFs.

The enhanced photocatalytic activity of Au/ZnO NFs can be explained based on the role of metal nanoparticles in modulating the electronic band structure of metal oxide semiconductors, as illustrated in Figure 6e,f. Because the halogen lamp contains a weak near-UV component near 380–400 nm, the observed photocatalytic activity under halogen-lamp illumination may include partial excitation of ZnO by residual near-UV photons. The photogenerated electron in the conduction band (CB) can be captured by dissolved oxygen (O_2_) to form superoxide radical anions (O_2_^•–^), which subsequently react with water to generate hydroxyl radicals (^•^OH).^49–51^ Meanwhile, photogenerated holes in the valence band (VB) can be trapped by surface hydroxyl groups, also leading to the formation of highly reactive ^•^OH. These reactive species then attack and decompose organic dye molecules present at or near the ZnO surfaces. However, photogenerated electrons and holes can easily recombine, reducing the number of charge carriers available for photocatalytic reactions and thus decreasing the photocatalytic efficiency. Moreover, the relatively low light intensity near 380–400 nm results in a limited rate of electron–hole generation, which can further slow the degradation of dye molecules. In addition, the photodegradation of MB using ZnO and Au/ZnO NFs catalysts under UV-light irradiation was also investigated, as shown in Figure S7.

By decorating ZnO NFs with Au nanoparticles, LSPR can be induced under near-UV and visible-light illumination. The generated hot electrons may be transferred to the CB of ZnO, leading to enhanced formation of reactive oxygen species. In addition, photogenerated electrons in the CB of ZnO can be transferred to the Au nanoparticles, which act as electron sinks, thereby suppressing electron–hole recombination. The remaining holes in the VB of ZnO further contribute to photocatalytic oxidation reactions. Additionally, plasmon-generated hot electrons facilitate the reduction of dissolved O_2_ to O_2_^•–^, which subsequently undergoes secondary reactions leading to the formation of reactive ^•^OH species. As a result, the Au/ZnO NFs exhibit greatly enhanced photocatalytic degradation of MB compared with pristine ZnO.

## 4 Conclusions

In summary, a hierarchical photocatalyst featuring defect states and SPR effects was successfully synthesized through the decoration of Au nanoparticles on ZnO NFs, resulting in significantly enhanced photocatalytic performance compared with pristine ZnO NFs. Structural and compositional analyses reveal the preservation of the ZnO NF morphology and the uniform decoration of Au nanoparticles on the ZnO surface. Optical characterizations indicate that Au decoration introduces strong visible-light absorption via LSPR without affecting the intrinsic band gap of ZnO. Decoration with Au nanoparticles significantly quenches the NBE and defect level emissions of ZnO NFs, as revealed by PL, PLE, and TRPL analyses. This behavior indicates plasmon-induced hot-electron generation, enhanced interfacial electron transfer, and suppressed electron–hole recombination through efficient electron trapping. These effects significantly prolong charge-carrier lifetimes and promote effective charge separation. The synergistic interaction between plasmonic Au nanoparticles and ZnO NFs leads to increased formation of reactive oxygen species under visible-light irradiation. In addition, superior visible-light absorption is facilitated by the hierarchical structure, which provides a large surface area and an increased number of catalytically active sites. As a result, the Au/ZnO NF catalysts achieve nearly complete dye degradation within a significantly shorter irradiation time compared with pristine ZnO. This work offers a promising strategy for designing efficient visible-light-driven photocatalysts for wastewater treatment and environmental remediation.

## Supplementary Material



## Notes

Fitting parameters of TRPL decay curves for pristine ZnO and Au/ZnO NFs; EDS spectra of pristine ZnO and Au/ZnO NFs; TEM images of pristine ZnO and Au/ZnO NFs; *I*–*V* characteristics of Au/ZnO NFs measured under dark and light-illumination conditions; emission spectrum of the halogen lamp; MB degradation under light irradiation without catalyst and in the presence of catalysts under dark conditions; photocatalytic degradation of methylene blue using Au/ZnO catalysts with different Au loading amounts; photocatalyst performance of pristine ZnO and Au/ZnO NFs for MB degradation under UV-light illumination (PDF)
