# Supplementary material for: Photoluminescence Excitation Engineering of Gold Nanoparticle-Decorated Zinc Oxide Nanoflowers for Efficient Visible Photocatalytic Water Treatment
Source: ACS Omega. 2026 Jun 16;11(25):38134–43. doi: 10.1021/acsomega.6c03907 (PMC13325363; doi:10.1021/acsomega.6c03907)
Supplement: Supplementary file 1 [file ao6c03907_si_001.pdf]

Supporting Information

Photoluminescence Excitation Engineering of Gold Nanoparticle-Decorated Zinc Oxide Nanoflowers for Efficient Visible Photocatalytic Water Treatment

Siwaporn Khemphet,[a,b] Sayan Pudwat,[a,b] Nattasamon Petchsang,[c] Yong-Hoon Kim,[d] Tanakorn Osotchan,[e] Pairote Jaideaw,[f] Nopporn Poolyarat,[g] Rawat Jaisutti[a,b*]

[a]Department of Physics, Faculty of Science and Technology, Thammasat University, Pathum Thani 12120, Thailand

[b]Research Unit in Innovative Sensors and Nanoelectronic Devices, Thammasat University, Pathum Thani 12120, Thailand

[c]Department of Materials Science, Faculty of Science, Kasetsart University, Bangkok, 10900, Thailand

[d]School of Advanced Materials Science and Engineering, Sungkyunkwan University, Suwon 16419, Korea

[e]Department of Physics, Faculty of Science, Mahidol University, Bangkok 10400, Thailand

[f]Physics and General Science Program, Faculty of Science and Technology, Nakhon Ratchasima Rajabhat University, Nakhon Ratchasima 30000, Thailand

[g]Thailand Institute of Nuclear Technology, Nakhon Nayok 26120, Thailand

Corresponding Author: jrawat@tu.ac.th

Table S1. Fitting parameters of TRPL decay curves for pristine ZnO and Au/ZnO nanoflowers with various Au loading (wt%).

Sample	τ_1 (ns)	A_1	τ_2 (ns)	A_2
ZnO	1.12	0.8857	8.99	0.2086
Au5/ZnO	1.04	1.0405	10.39	0.1319
Au10/ZnO	1.03	1.0844	11.07	0.1155
Au15/ZnO	0.95	1.1046	10.87	0.1020

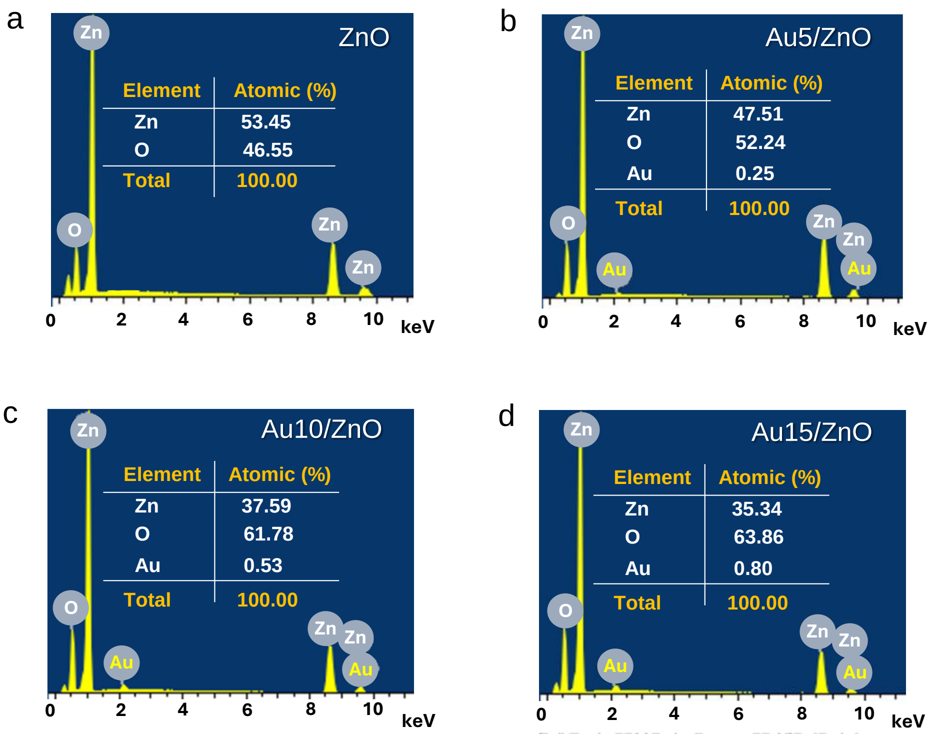

Figure S1. EDS spectra of pristine (a) ZnO, (b) Au5/ZnO, (b) Au10/ZnO and (d) Au15/ZnO nanoflowers.

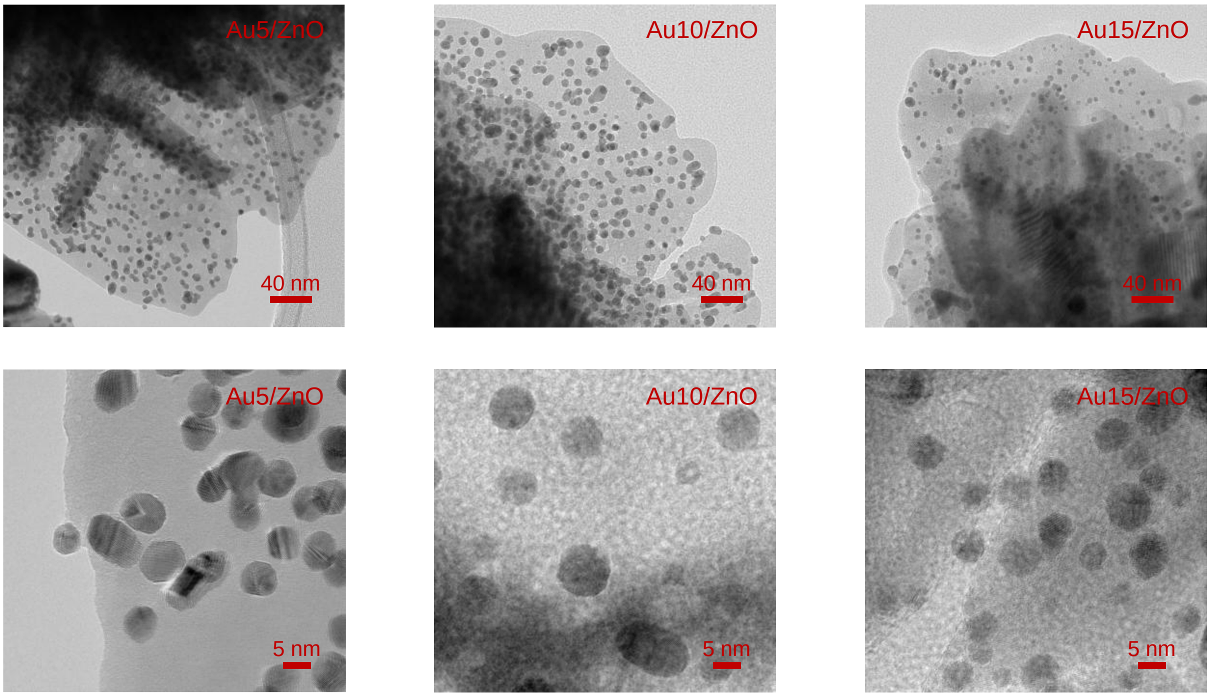

Figure S2. TEM/HRTEM images of Au/ZnO nanoflowers with varying Au loading amounts.

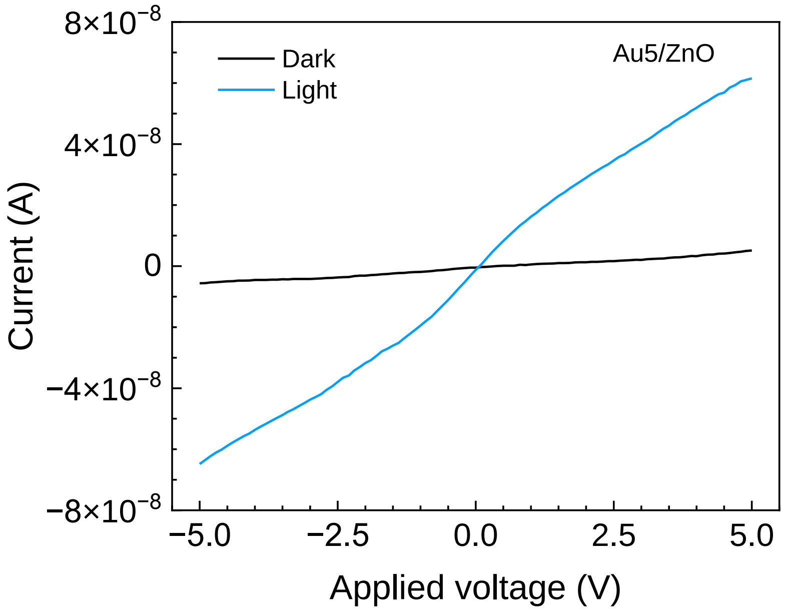

Figure S3. I-V characteristics of Au5/ZnO NFs measured under dark and light-illumination conditions.

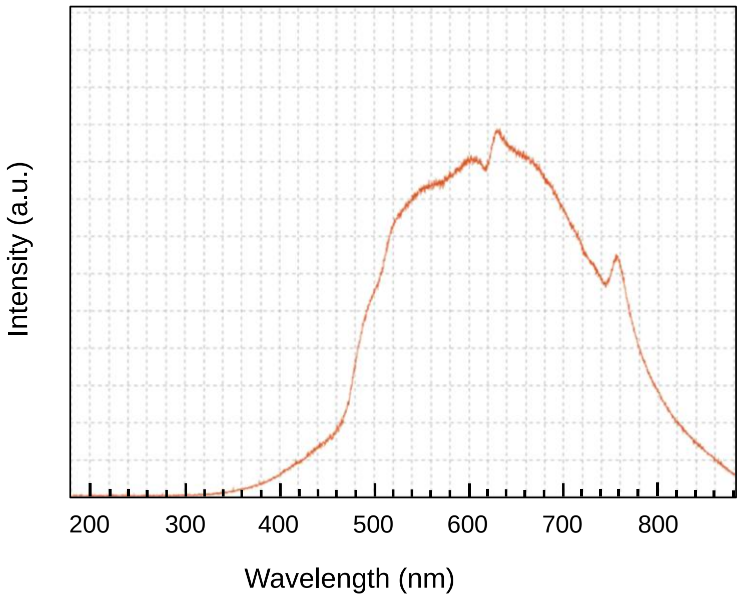

Figure S4. Emission spectrum of the halogen lamp (50 W low-voltage halogen lamp, Philips) measured using a USB4000 spectrometer (Ocean Optics).

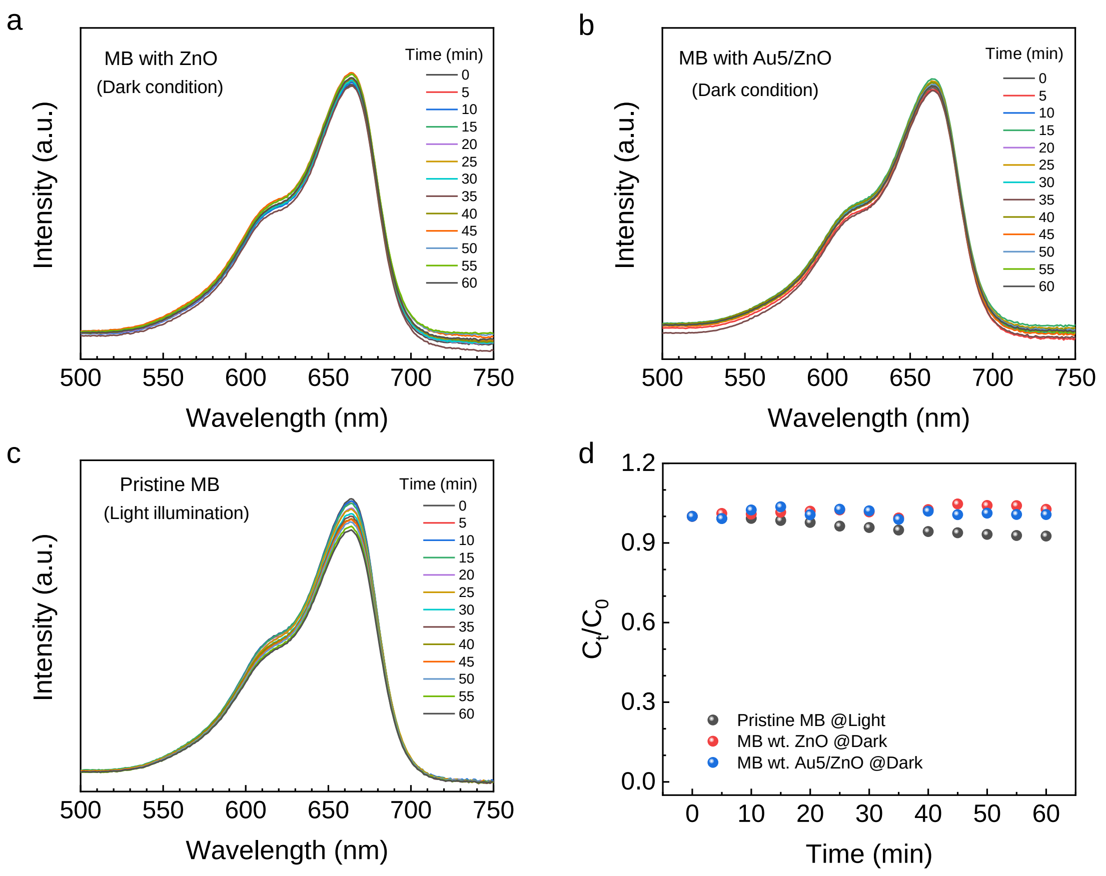

Figure S5. Methylene blue degradation in the presence of (a) ZnO and (b) Au5/ZnO catalysts under dark conditions, and (c) under visible-light irradiation without a catalyst. (d) Normalized degradation ratio plots as a function of irradiation time.

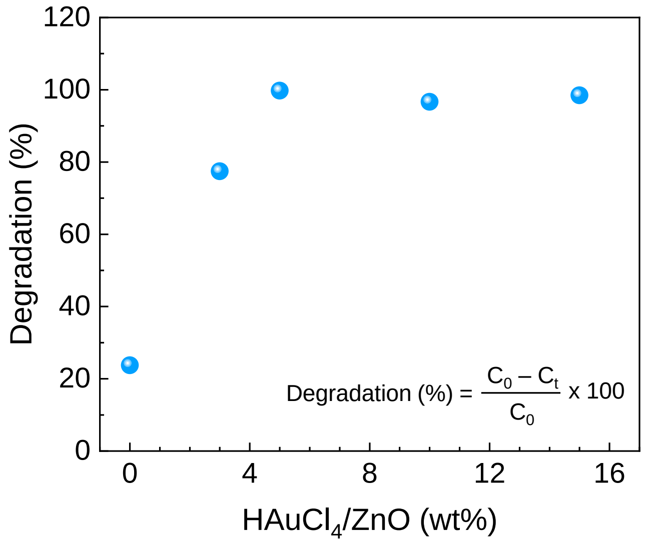

Figure S6. Photocatalytic degradation of methylene blue using Au-loaded ZnO nanoflowers with different Au loading amounts under visible-light illumination.

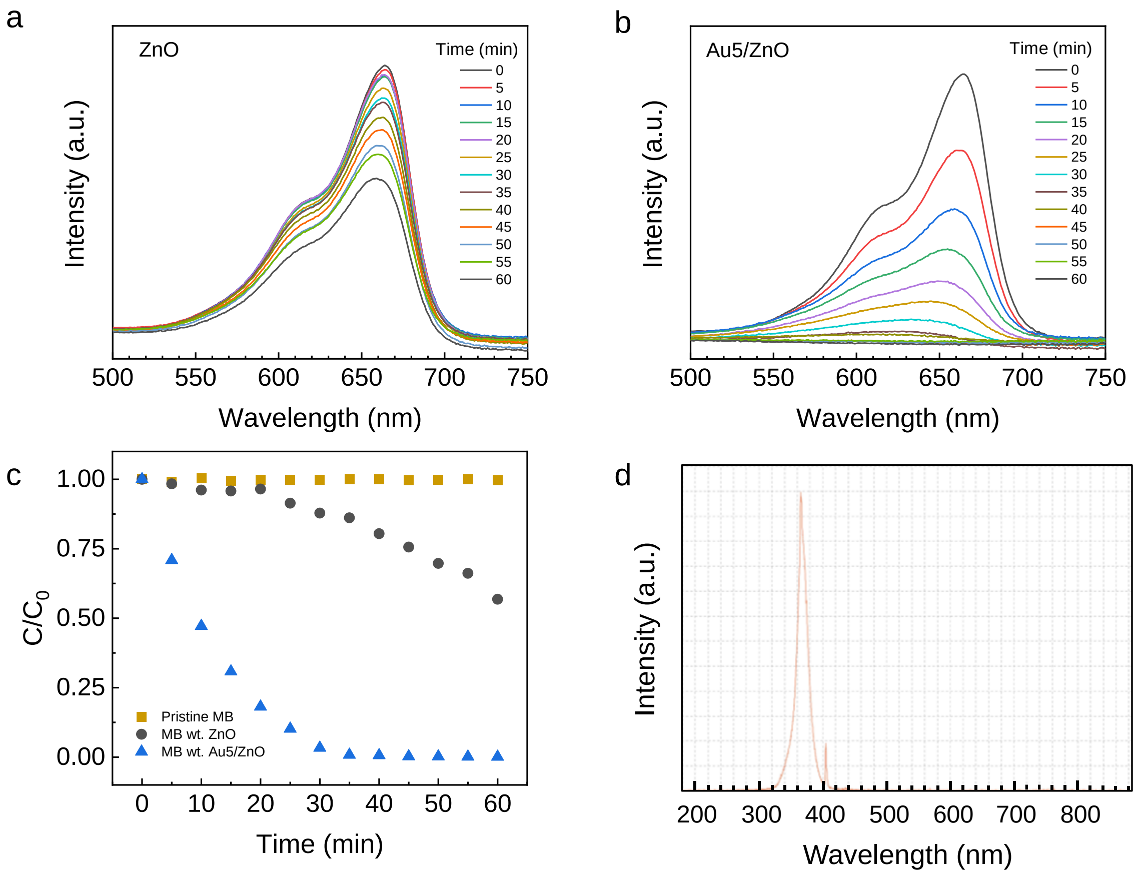

Figure S7. Photocatalyst performance of pristine ZnO and Au/ZnO nanoflowers for methylene blue degradation under UV light illumination. UV-Vis absorption spectra of methylene blue solution in the presence of (a) ZnO and (b) Au5/ZnO photocatalysts. (c) Normalized degradation ratio plots as a function of irradiation time. (d) Emission spectrum of the UV light source measured using a USB4000 spectrometer (Ocean Optics).
